# From Offshore to Onshore: Multiple Origins of Shallow-Water Corals from Deep-Sea Ancestors

**DOI:** 10.1371/journal.pone.0002429

**Published:** 2008-06-18

**Authors:** Alberto Lindner, Stephen D. Cairns, Clifford W. Cunningham

**Affiliations:** 1 Centro de Biologia Marinha, Universidade de São Paulo, São Sebastião, São Paulo, Brazil; 2 National Museum of Natural History, Smithsonian Institution, Washington, D. C., United States of America; 3 Biology Department, Duke University, Durham, North Carolina, United States of America; American Museum of Natural History, United States of America

## Abstract

Shallow-water tropical reefs and the deep sea represent the two most diverse marine environments. Understanding the origin and diversification of this biodiversity is a major quest in ecology and evolution. The most prominent and well-supported explanation, articulated since the first explorations of the deep sea, holds that benthic marine fauna originated in shallow, onshore environments, and diversified into deeper waters. In contrast, evidence that groups of marine organisms originated in the deep sea is limited, and the possibility that deep-water taxa have contributed to the formation of shallow-water communities remains untested with phylogenetic methods. Here we show that stylasterid corals (Cnidaria: Hydrozoa: Stylasteridae)—the second most diverse group of hard corals—originated and diversified extensively in the deep sea, and subsequently invaded shallow waters. Our phylogenetic results show that deep-water stylasterid corals have invaded the shallow-water tropics three times, with one additional invasion of the shallow-water temperate zone. Our results also show that anti-predatory innovations arose in the deep sea, but were not involved in the shallow-water invasions. These findings are the first robust evidence that an important group of tropical shallow-water marine animals evolved from deep-water ancestors.

## Introduction

The discovery of high species richness in the deep sea has led to a burst of hypotheses aiming to explain the origin and evolution of marine biodiversity [1]–[3]. Ecological explanations for high species diversity in deep waters include, among others, time-stability [1] and habitat heterogeneity [2], whereas evolutionary explanations have been articulated on the basis of the long-proposed hypothesis that deep-water fauna originated from shallow-water ancestors [3], [4]. For example, a shallow (onshore) to deep (offshore) evolutionary pattern has been invoked to explain the evolution of the Phanerozoic marine fauna [4], for abyssal taxa in general [5], [6], and for the origin of hydrothermal vent communities [7]. An onshore to offshore pattern of marine evolution has also been proposed for a number of particular taxa, including groups of mollusks, crustaceans, and echinoderms, among others (e.g., [8]–[11]). Possible explanations for the general onshore-offshore evolutionary pattern include a preferential origin of lineages in shallow waters due to anoxic events in the deep sea before the late Cretaceous [12] and the displacement of lineages into the deep sea as a result of high predation in shallow waters [13].

Yet, detailed analyses of the fossil record also indicate that a shallow/onshore to deep/offshore pattern of evolution only holds for the initial diversification of the major groups of marine invertebrates, with ‘major’ referring to groups so distinct that they are assigned a taxonomic rank of ‘Order’ [14], [15]. Following the initial onshore-offshore diversification, subsequent evolution of lineages may also have proceeded from offshore to onshore. The fossil record also indicates that important groups of animals that presently thrive in both deep and shallow waters—such as neogastropod mollusks and stylasterid corals—may have originated in deep offshore environments [14], [15].

**Figure 1:**
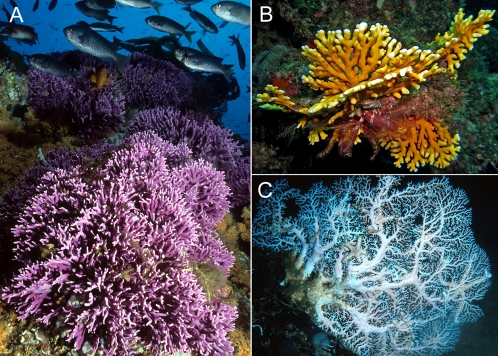
Stylasterid corals. (A) The shallow-water California hydrocoral *Stylaster californicus*, United States of America. (B) Tropical shallow-water species of *Distichopora*, Papua New Guinea. (C) Deep-water species of *Stylaster*, 536 meters depth, St. Lucia. Images courtesy of R Morse (A), M Baine (B), JK Reed (C).

In this study, we used a worldwide phylogeny of stylasterid corals (Cnidaria: Hydrozoa: Stylasteridae) to test the hypothesis that they originated in the deep sea. Stylasterid corals are the second most diverse group of extant hard corals and an abundant component of marine communities in shallow and deep waters (Figure 1). Also known as ‘lace corals’ or ‘hydrocorals’, the 250 described stylasterid species represent ∼15% of hard coral diversity [16] and share with scleractinian corals (Cnidaria: Anthozoa: Scleractinia, the most species rich group of hard corals) the evolutionary innovation of a rigid skeleton of calcium carbonate. Stylasterid corals are known since the Paleocene [14], [15], [17] and are currently found from the intertidal zone to depths of up to 2800 meters [18]. Our results not only support earlier suggestions from the fossil record that stylasterid corals first appeared in a deep-water setting, but reveal that this group of corals has diversified extensively in the deep sea and invaded the shallow-water tropics three times and the temperate zone once.

## Results

**Figure 2:**
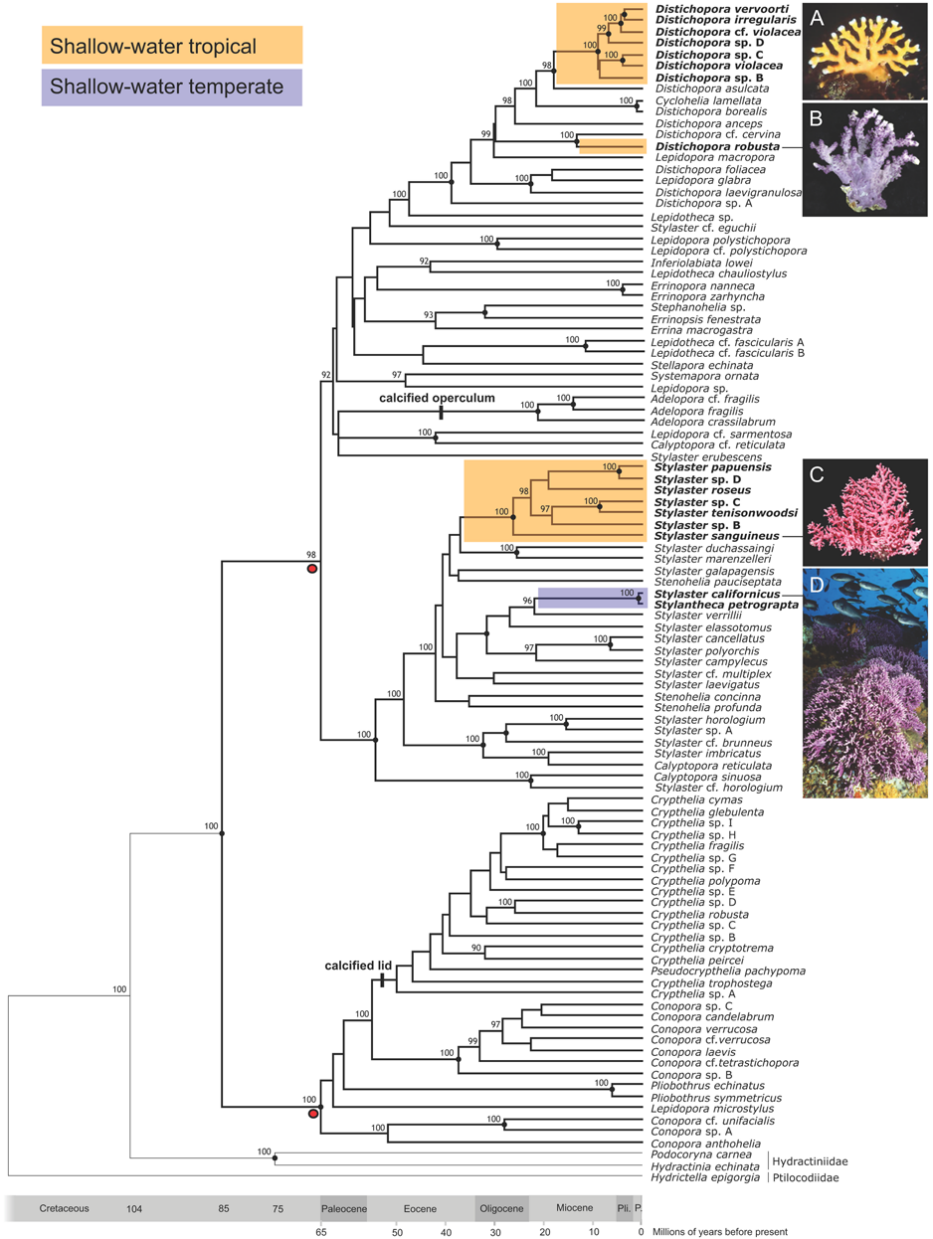
Phylogenetic relationships of stylasterid corals and multiple invasions of shallow waters. (A) Tropical Indo-West Pacific species of *Distichopora*. (B) Tropical Eastern Pacific species *Distichopora robusta*. (C) Circumtropical species of *Stylaster*. (D) Temperate North Pacific species *Stylantheca petrograpta* and *Stylaster californicus.* Remaining stylasterid species are deep-sea dwellers. The tree was estimated by maximum likelihood (ML) using a 2638 bp DNA dataset (−ln likelihood 25390.87513). Character state reconstructions estimate the ancestral environment of stylasterid corals as deep water (probabilities of 0.98 and 0.97 using Mk1 and AsymmMk models, respectively [43]). The hypothesis of a single invasion of shallow waters is rejected (p<0.001, Shimodaira-Hasegawa test [46]). Nodes marked with red circles are calibrated using the earliest stylasterid fossils, 65 mya. Numbers are Bayesian posterior probabilities whenever >90%; dots at nodes indicate bootstrap support values whenever >70% for both parsimony and maximum likelihood. Images courtesy of B Hoeksema (A), SD Cairns (B), PMS Peres (C), R Morse (D).

To infer the evolutionary relationships among shallow-water and deep-sea stylasterid corals we used 2638 base pairs of aligned molecular dataset (mitochondrial 16S rDNA and nuclear 18S rDNA and calmodulin genes) obtained for 100 species (in 20 of the 26 genera) collected worldwide (Table S1). These 100 species represent 36% of the 276 known stylasterid coral species (250 described species and 26 new species reported herein in Table S1). The phylogeny shows that stylasterid corals originated and diversified in the deep sea, and invaded the shallow-water tropics three times (Figure 2). Our phylogeny was inferred using maximum likelihood and is corroborated by Bayesian analyses (Figure 2).

The hypothesis that stylasterid corals originated in deep waters is strongly supported by maximum-likelihood character state reconstructions (Figure 2). The common ancestor of all stylasterid corals is reconstructed as deep water with a probability >0.96. Likewise, the immediate ancestors of shallow-water clades—one temperate and three tropical—and their closest deep-water relatives are all reconstructed as deep water with probabilities >0.97 (clades A–D in Figure 2). The two most species rich clades of tropical shallow-water stylasterids—the Indo-West Pacific species of *Distichopora* (Figure 2A) and the circumtropical species of *Stylaster* (Figure 2C)—form well supported monophyletic groups.

The phylogenetic hypothesis of a deep to shallow-water pattern of diversification is further supported by the fossil record, which indicates that stylasterid corals first appeared in a deep-water setting in the lower Paleocene, 65 mya (Table S2) [14], [15], [17]. To investigate the possible role of predation on stylasterid coral diversification, we reconstructed the evolutionary history of their two most conspicuous defensive features—the calcified lids and opercula that protect feeding polyps of *Crypthelia* and *Adelopora* [19], respectively. The phylogeny supports the hypothesis that both structures evolved in the deep sea (Figure 2).

As part of our sampling efforts we discovered 26 undescribed stylasterid species, which we used in phylogenetic analyses. Twenty-five of these new species were found on seamounts in the Norfolk Ridge off New Caledonia, Southwest Pacific (Table S1).

## Discussion

Our results are the first demonstration of multiple invasions of tropical shallow waters by a major group of benthic marine invertebrates previously restricted to offshore, deep-water environments. Although the fossil record suggested that stylasterid corals are one of the few groups of well-preserved marine invertebrates with an offshore origin [14], [15], [17], we find that shallow waters have been invaded at least four times, once into shallow temperate waters, and three times into the shallow-water tropics (Figure 2). This result reveals that the shallow-water tropics are not only a source of diversity [20], but have accumulated species and lineages from the deep sea. The phylogenetic results also show that the two most species rich clades of tropical shallow-water stylasterids—the Indo-West Pacific species of *Distichopora* (Figure 2A) and the circumtropical species of *Stylaster* (Figure 2C)—form distinct, well-supported monophyletic groups.

Our conclusion that colonization of shallow waters has happened relatively recently is strengthened by the extensive sampling of shallow-water taxa, making it unlikely that basal shallow-water clades were missed in our analysis. Specifically, we sampled 10 of the 24 described shallow-water stylasterid species (42%) plus an additional seven possibly undescribed species (Figure 2; see Table S1 for a list of species and notes on taxonomy). Moreover, we found species of both tropical shallow-water stylasterid genera—*Distichopora* and *Stylaster*—from around the world, including the Atlantic Ocean, the Eastern Pacific, and the Indo-West Pacific. The unsampled species of tropical shallow-water stylasterids are morphologically similar to the shallow-water *Distichopora* clade A and *Stylaster* clade C (Figure 2). The only shallow-water genus that could not be sampled in this study is the cold-water *Gyropora*, with a single species found in southern South Africa [21]. This genus is morphologically similar to *Distichopora* and *Errinopora*, and may represent an additional invasion of shallow waters.

**Figure 3:**
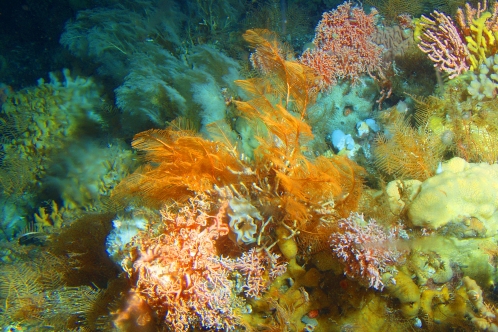
Diverse benthic habitat off Adak Island, Alaska, showing sponges, hydroids, and stylasterid corals of the genus *Stylaster* at a depth of 150 meters. Stylasterid corals are among the most important habitat-forming invertebrates in deep-sea coral ecosystems in the North Pacific [24]. Photo by Alberto Lindner/NOAA.

Corals are best known to thrive in warm shallow waters, but almost 66% of coral diversity (3,336 of the 5,080 known coral species) is found in waters deeper than 50 meters [16]. Percentages of deep-water coral species are greater for stylasterid corals (89%), black corals (75%), and octocorals (75%), but as much as 41.5% of the 1482 living scleractinian corals (the major group of hard corals) are also found exclusively in the deep sea [16]. Both shallow-water and deep-sea scleractinians are known since the Mesozoic [14], [15], [22], and it is possible that deep-water scleractinians, black corals, and octocorals, have also contributed to the shallow-water marine communities. It is noteworthy that recent evidence suggests deep-sea coral ecosystems may compare in species richness and abundance to shallow-water tropical reefs (Figure 3). These deep-sea coral ecosystems have been best studied in waters 150–1000 meters deep in the North Atlantic (e.g., [23]) and, more recently, in the North Pacific (e.g., [24]). Although the diversity and ecology of deep-water coral habitats is still poorly investigated, it is possible that, in contrast to the possibly more stable deep-sea abyss, such upper bathyal (150–1000 meters deep) coral ecosystems are highly dynamic environments that provide opportunities for the evolution and diversification of taxa that subsequently invade other regions, including the shallow-water tropics.

Within the Stylasteridae, the evolutionary processes that have led to the deep to shallow-water pattern of evolution remain elusive, but our results indicate that sculptured defensive innovations in stylasterid corals, such as lids and opercula that protect feeding polyps, evolved in the deep sea (Figure 2). Evolution of these defensive innovations in deep waters and evidence of predation or parasitism on deep-water stylasterids [19], [25] indicate that predator-prey interactions may have been important in shaping the evolution of these corals. This is some of the first evidence to suggest that predation may be an important evolutionary force not only in shallow waters [13], but also in the deep sea. The discovery of deep-sea origins of anti-predatory defenses is doubly ironic. First, these defenses were absent in all of the stylasterid lineages that successfully invaded the high-predation environment of the shallow-water tropics. Second, a lower level of predation in the deep sea has been invoked to explain why ancient lineages have been able to survive in this environment [13].

The phylogenetic analyses identify four distinct shallow-water lineages of stylasterid corals, that may provide information for conservation purposes. For example, our results show that the tropical Eastern Pacific, shallow-water species *Distichopora robusta* (Figure 2B), is not an offshoot of the Indo-West Pacific shallow-water *Distichopora* clade (Figure 2A), but evolved independently from deep-water ancestors. Similarly, the California hydrocoral *Stylaster californicus* is part of a shallow-water lineage (Figure 2D) that evolved independently from the major clade of tropical shallow-water species of *Stylaster* (Figure 2C). Protection of multiple lineages of shallow-water corals is an important goal in marine conservation [26], and plans to protect stylasterid corals should take into account the evolutionary distinctiveness of the four lineages that presently occur in shallow waters.

Our study reinforces the importance of large-scale phylogenetic analyses for understanding the evolution of marine animals, including corals [26]. Although it remains to be seen whether additional groups of marine organisms have diversified from deep to shallow-water environments, possible deep-water origins of other animal groups, such as neogastropod mollusks [14], [15] and reef fishes [27], suggest that the deep sea has contributed repeatedly to the formation of shallow-water communities. Tracking the history of these lineages in space and time should provide additional new insights into the contribution of deep-water environments to the evolution of marine biotas.

## Materials and Methods

### Taxon sampling

A worldwide sampling of 100 stylasterid species (classified in 20 of the 26 genera) was obtained during scientific expeditions conducted by the United States National Oceanic and Atmospheric Administration (NOAA) off the Gulf of Alaska (in 2001), the Aleutian Islands (in 2002), and Washington State (in 2003), as well as during the ‘Norfolk2’ expedition conducted by the L'Institut de recherché pour le développement (IRD), in 2003, off New Caledonia (Table S1). Specimens provided by collaborators or previously deposited at the United States National Museum of Natural History, Smithsonian Institution and at the Harbor Branch Oceanographic Institution were also used for molecular phylogenetic analyses (museum catalog numbers for voucher specimens are provided in Table S1). These collections provided representatives of deep-sea and shallow-water stylasterid species from the Atlantic Ocean (including the Caribbean), Eastern Pacific (including the Galápagos Islands), Subantarctic region, and the Indo-West Pacific. Such a broad taxonomic and geographic sampling was important to gather representatives from the major shallow-water and deep-sea stylasterid lineages.

### Identification of shallow-water and deep-sea stylasterid corals

The boundary between ‘shallow water’ and ‘deep sea’ for stylasterid corals was defined at a depth of 50 meters, as previously adopted for corals in general [16]. A 50 meter wave-disturbance boundary is used to determine the division between ‘onshore’ and ‘offshore’ environments in the fossil record [28]. This 50 meter boundary corresponds approximately to the maximum depth that normal storm waves reach to disturb the benthos significantly. Evidence of actual wave disturbance on the ocean bottom at a maximum depth of ∼50 meters include the ‘ripple scour depressions’ reported for depths of 45–60 meters off California [29]. Although strong storm waves may disturb the ocean floor up to 100 meters deep (A.B. Murray, personal communication), such waves are more comparable to the ‘maximum’ storm waves used as the division between middle and outer shelves within the offshore category [28].

We determined the mean depth distribution for the 100 species used in phylogenetic analyses using over 2000 specimen records from taxonomic revisions, museum catalogs, and newly collected specimens (Table S1). To compute mean depths, we used the number of stations in which a species was collected, regardless of the number of specimens collected at each station. This approach greatly reduced the number of specimen records for calculation of mean depths but was necessary since the number of specimens collected per station usually results from semi-quantitative collecting techniques, such as dredging.

Although we used a 50 meters depth boundary consistent with previous studies [16], our conclusions remain unchanged if we adopt a boundary of ∼130 meters, corresponding to the worldwide average depth of the continental shelf break, which sets the classic division between the shallow continental shelf and the deep sea (e.g., [30]). This includes the continental slope, rise, abyssal plain, trenches, and other deep-water features, such as seamounts. If a boundary of 130 meters is adopted, the only change in our results is that *Distichopora* cf. *cervina*, with a single occurrence at 73 meters (Table S1), would be considered a shallow-water dweller. Since *D.* cf. *cervina* is strongly supported as sister to the shallow-water *Distichopora robusta* (Figure 2), the number and topology of the shallow-water invasions (four) does not change regardless of whether the species is coded as ‘shallow-water’ or ‘deep-water’ dweller.

It is also important to note that a few deep-water stylasterid species, such as *Errina novaezelandiae*, are found in shallow-water fjords (e.g., [31]), a phenomenon known as deep-water emergence, i.e., the occurrence of deep-water species in some shallow-water regions, particularly in high latitudes where the water temperature is about equal at the surface and in deep waters. Since occurrence of deep-sea species in shallow-water fjords was possible only after the last glacial maximum (characterizing a recent and possibly transient range extension), and since most of the species range remains in the deep sea (rather than predominantly or exclusively in shallow waters) we do not consider deep-water emergence as a true shallow-water invasion.

### DNA extraction, amplification, and sequencing

Genomic DNA was extracted using standard techniques followed by ethanol precipitation. Branch portions ∼0.5–2.0 cm long were ground individually in liquid nitrogen or placed in 96-well plates for extraction. The three targeted gene fragments—large ribosomal subunit of the mitochondrial RNA (lsu-rRNA, 16S), small subunit of the nuclear RNA (ssu-rRNA, 18S), and nuclear calmodulin (CaM)—were amplified using polymerase chain reaction (PCR): 35 cycles with annealing temperatures of 50°C for the 16S gene, 58–64°C for the CaM gene and 58°C for the 18S gene, followed by a 5 minute extension phase at 72°C. Previously published primers were used to amplify and sequence the 16S marker [32] and the 18S gene [33].

We developed primers for amplification and sequencing of the CaM gene (352 bp exon) on the basis of an unpublished calmodulin sequence from the hydractiniid *Podocoryna carnea* provided by J. Spring (University of Basel) and the anthozoan *Metridium senile* [34]: **forward primers** CAMF1 (General Hydrozoa) 5′-GATCAAYTRACNGARGAACAAATTGC-3′ and CAMF2 (Hydractinoidea-specific) 5′-CAATTGACTGAGGAACAAATTGC-3′ and **reverse primers** CAMR1 (General Hydrozoa) 5′-CCATCNCCATCRATATCAGC-3′, CAMR2a (General Hydrozoa) 5′-TTGGACATCATCATYTTNACRAACT-3′, and CAMR2b (Hydractinoidea-specific) 5′-TGGACATCATCATTTTCACGA-3′. Usage of reverse primers CAMR2a and CAMR2b also yields an intron at the 3′ end of the CaM exon, which was not used for phylogenetic analyses due to drastic differences in size among species.

PCR products were sequenced directly in both directions and heterozygous copies of the nuclear gene CaM were detected as double peaks using the software Sequencher 4.1.2 (Gene Codes Corporation) and coded as ambiguous. DNA sequences for outgroup species were obtained from previous studies [35], [36]. These species are *Bougainvillia* sp. and three species of Hydractinoidea, the superfamily that also embraces the family Stylasteridae: *Hydrictella epigorgia* (Ptilocodiidae) and *Hydractinia echinata* and *Podocoryna carnea* (Hydractiniidae). The family Hydractiniidae has been previously shown to be the sister group of the Stylasteridae based on both morphological and molecular phylogenetic data [21], [36]. The combined 16S, CaM and 18S alignment, totaling 2691 DNA base pairs (bp), was edited by eye and 53 bp corresponding to ambiguous regions within the 16S and 18S fragments were excluded, resulting in a final alignment of 2638 bp. DNA sequences generated in this paper are available in GenBank (accession numbers EU645266-EU645479).

### Phylogenetic analyses

Phylogenetic analyses were performed in PAUP* [37] using parsimony and maximum likelihood with a GTR+Γ+I model of DNA substitution as determined by the hierarchical likelihood ratio test implemented in Modeltest [38]. The appropriate PAUP settings and model parameters used in the maximum likelihood analysis are as follows: Lset Base = (0.344945 0.152759 0.211326) Nst = 6 Rmat = (4.21232 10.65315 4.94139 1.15379 30.05541) Rates = gamma Shape = 0.647751 Pinvar = 0.701636. We evaluated node support using 1000 non-parametric bootstrap pseudoreplicates for parsimony and 100 pseudoreplicates for maximum likelihood. Bayesian analyses were performed with estimation of model parameters unlinked in MrBayes [39], i.e., unlinking 16S, CaM, and 18S datasets. Bayesian analyses were run for 2 million generations with trees sampled every 500 generations. An 85% majority-rule consensus was performed on the sampled trees (except burnin trees) in PAUP.

### Rate smoothing and temporal calibration

The most likely tree was transformed into a chronogram using the first records of stylasterids, 65 mya, as calibrations for the two most basal stylasterid nodes (marked with red dots in Figure 2) in r8s [40] (see Table S2 for a list of stylasterid fossils), following deletion of the most distant outgroup, *Bougainvillia* sp., as required by r8s [40]. Calibration points were provided by the earliest known stylasterid fossils, from the 65 mya Fakse formation [17]. This first assemblage is already morphologically diverse, including species that can be unambiguously assigned to each of the two major stylaterid clades, marked with red dots in Figure 2.

Fossil calibration for Clade 1: The fossil *Conopora arborescens* from the Fakse formation has double-chambered gastropore tubes [17], a character shared by all members of the clade including *Conopora* and *Crypthelia*. With the exception of *Pseudocrypthelia pachypoma*, all species in this clade also lack gastrostyles, a feature also lacking in the fossil *Conopora arborescens* [17].

Fossil calibration for Clade 2: In contrast to *Conopora arborescens*, the Fakse formation fossil *Errina lobata* has gastrostyles and lacks a double-chambered gastropore tube. This combination of characters characterizes almost all the taxa in the second major clade of extant stylasterids, the clade containing *Distichopora* and *Stylaster.*

Because both major clades had already appeared by 65 mya, the divergence between the clades must have happened even earlier. For this reason, the most recent common ancestor of each clade was fixed at 65 mya, instead of placing the 65 mya calibration at the common ancestor of all stylaterids (see Figure 2). It is important to emphasize that, because these 65 mya fossils provide minimum-age estimates for the two major clades of stylasterid corals (marked with red dots in Figure 2), the actual ages of the shallow-water invasions may be older than presented in Figure 2. In fact, if substitution rates for the 16S gene are calculated on the basis of the 65 mya fixed nodes, the resulting values are considerably greater than those previously calculated for other hydrozoans. For example, 16S rates calculated based on the branches leading to *Conopora anthohelia* and *Stylaster* cf. *horologium* (Figure 2) are on the order of 7.71×10^−9^ and 4.49×10^−9^ substitutions site^−1^ year^−1^, respectively. These rates are 2 to 6 times faster than those inferred for hydractiniid and campanulariid hydrozoans (1.25×10^−9^ and 2.44×10^−9^ substitutions site^−1^ year^−1^, respectively [41]), suggesting that stylasterids either have faster rates of 16S substitution than other hydrozoans or that the age of both major clades marked with red dots in Figure 2 are in fact older than 65 mya.

The inferred chronogram (Figure 2) was used for ancestral character state reconstructions using both the Mk1 [42] and the AsymmMk options in Mesquite [43], which assume a stochastic model of evolution [44], [45]. The monophyly of shallow-water clades was examined with the Shimodaira-Hasegawa (SH) test [46] using the RELL test distribution in PAUP.

## Supporting Information

Table S1 Geographic and bathymetric distributions of the 100 species used in phylogenetic analyses, and GenBank accession numbers and Museum catalog numbers.
(0.12 MB DOC)

Table S2 Fossil stylasterid species and paleontological information.
(0.10 MB DOC)
