# Supplementary material for: From Offshore to Onshore: Multiple Origins of Shallow-Water Corals from Deep-Sea Ancestors
Source: PLoS One. 2008 Jun 18;3(6):e2429. doi: 10.1371/journal.pone.0002429 (PMC2424010; doi:10.1371/journal.pone.0002429)
Supplement: Table S1 — Geographic and bathymetric distributions of the 100 species used in phylogenetic analyses, and GenBank accession numbers and Museum catalog numbers. (0.12 MB DOC) [file pone.0002429.s001.pdf]

**Table S1.** Geographic and bathymetric distributions of the 100 species used in phylogenetic analyses, and GenBank accession numbers and Museum catalog numbers.

| Species[a] | Geographic Distribution | Bathymetric Distribution (in meters)[b] Mean [range] (n) | % Records Shallow (<50m) | % Records Deep (>50m) | GenBank Accession Number 16S | CaM | 18S | Catalog number of specimen used in phylogenetic analyses | Source of distributional records and taxonomy[c] |
|---|---|---|---|---|---|---|---|---|---|
| *Pliobothrus echinatus* | West and North Atlantic | **443** [165-750] (6) | | 100 | EU645266 | EU645366 | EU645428 | H22-VIII-01-2-N-e | [1] |
| *Pliobothrus symmetricus* | West Atlantic | **440** [139-1100] (54) | | 100 | EU645267 | EU645367 | - | USNM77121 | [1-3] |
| *Conopora anthohelia* | Southwest Pacific | **737** [440-1175] (3) | | 100 | EU645268 | EU645368 | EU645429 | USNM1027755 | [4] |
| *Conopora* n. sp. cf. *unifacialis* | Southwest Pacific | **717** [708-727] (2) | | 100 | EU645269 | EU645369 | EU645430 | USNM1027752 | ps |
| *Conopora* n. sp. A | Southwest Pacific | **727** (1) | | 100 | EU645270 | - | - | USNM1071853 | ps |
| *Conopora* n. sp. C | Southwest Pacific | **240** (1) | | 100 | EU645271 | EU645370 | EU645431 | USNM1027759 | ps |
| *Conopora laevis* | Indo-West Pacific | **510** [130-1052] (36) | | 100 | EU645272 | - | - | USNM1071854 | [4,5] |
| *Conopora verrucosa* | Circumpolar off Antarctica; off South America; Southwest Pacific | **815** [198-2626] (78)[d] | | 100 | EU645273 | - | - | USNM60036 | [4-6] |
| *Conopora* n. sp. cf. *verrucosa* | Southwest Pacific | **775** (1) | | 100 | EU645274 | - | - | USNM1071855 | ps |
| *Conopora candelabrum* | Southwest Pacific | **607** [240-1265] (22) | | 100 | EU645275 | EU645371 | EU645432 | USNM1027747 | [4] |
| *Conopora* n. sp. cf. *tetrastichopora* | Southwest Pacific | **506** [290-748] (6) | | 100 | EU645276 | - | - | USNM1071856 | ps |
| *Conopora* n. sp. B | Southwest Pacific | **801** [715-925] (4) | | 100 | EU645277 | - | - | USNM1071857 | ps |
| *Crypthelia trophostega* | North Pacific | **420** [150-1913] (16) | | 100 | EU645278 | EU645372 | EU645433 | USNM1075952 | [7] |
| *Crypthelia* n. sp. A | Southwest Pacific | **499** (1) | | 100 | EU645279 | - | - | USNM1071896 | ps |
| *Pseudocrypthelia pachypoma* | West and Southwest Pacific | **657** [430-1089] (5) | | 100 | EU645280 | EU645373 | EU272643 | USNM1027728 | [4,8,9] |
| *Crypthelia cryptotrema* | Southwest Pacific | **340** [180-474] (8) | | 100 | EU645281 | EU645374 | EU272641 | USNM1027758 | [5] |
| *Crypthelia peircei* | West Atlantic | **406** [89-838] (16) | | 100 | EU645282 | - | - | USNM94728 | [1] |
| *Crypthelia glebulenta* | Galápagos Islands | **502** [169-806] (4) | | 100 | EU645283 | - | - | USNM84763 | [10,11] |
| *Crypthelia cymas* | Galápagos Islands and Southwest Pacific | **523** [169-1097] (14) | | 100 | EU645284 | - | - | USNM84770 | [4,10,11] |
| *Crypthelia* n. sp. H | Southwest Pacific | **358** [310-406] (2) | | 100 | EU645285 | - | - | USNM1071858 | ps |
| *Crypthelia* n. sp. G | Southwest Pacific | **325** [240-474] (5) | | 100 | EU645286 | - | - | USNM1071859 | ps |
| *Crypthelia fragilis* | Southwest Pacific | **1297** [648-2316] (9) | | 100 | EU645287 | - | - | USNM1071860 | [4,6] |
| *Crypthelia* n. sp. I | Southwest Pacific | **598** [587-609] (2) | | 100 | EU645288 | - | - | USNM1071861 | ps |
| *Crypthelia* n. sp. F | Southwest Pacific | **310** (1) | | 100 | EU645289 | - | - | USNM1071862 | ps |
| *Crypthelia* n. sp. E | Southwest Pacific | **216** [192-240] (2) | | 100 | EU645290 | - | - | USNM1071895 | ps |
| *Crypthelia polypoma* | Southwest Pacific | **541** [290-927] (11) | | 100 | EU645291 | - | - | USNM1071864 | [4] |
| *Crypthelia* n. sp. B | Southwest Pacific | **707** [440-833] (5) | | 100 | EU645292 | - | - | USNM1071866 | ps |
| *Crypthelia* n. sp. C | Southwest Pacific | **393** [290-500] (4) | | 100 | EU645293 | - | - | USNM1071869 | ps |
| *Crypthelia* n. sp. D | Southwest Pacific | **555** [422-748] (4) | | 100 | EU645294 | - | - | USNM1071871 | ps |
| *Crypthelia robusta* | Southwest Pacific | **506** [388-709] (5) | | 100 | EU645295 | - | - | USNM1071874 | [4] |
| *Calyptopora sinuosa* | Southwest Pacific | **474** [142-762] (10) | | 100 | EU645296 | EU645375 | EU645434 | USNM1027751 | [4] |

## **Table S1.** (continued)

| **Species**[a] | **Geographic Distribution** | **Bathymetric Distribution (in meters)**[b] **Mean** [range] (n) | **% Records** **Shallow** (<50m) | **Deep** (>50m) | **GenBank Accession Number** **16S** | **CaM** | **18S** | **Catalog number of specimen used in phylogenetic analyses** | **Source of distributional records and taxonomy**[c] |
|---|---|---|---|---|---|---|---|---|---|
| *Calyptopora reticulata* | Southwest Pacific | **789** [93-2055] (40) | | 100 | EU645297 | - | - | USNM1071879 | [4,6] |
| *Calyptopora* n. sp. cf. *reticulata* | Southwest Pacific | **367** [240-440] (6) | | 100 | EU645298 | EU645376 | EU645435 | USNM1027723 | ps |
| *Stylaster* n. sp. cf. *horologium* | Southwest Pacific | **354** (1) | | 100 | EU645299 | EU645377 | EU645436 | USNM1027745 | ps |
| *Stylaster* n. sp. cf. *brunneus* | Southwest Pacific | **240** (1) | | 100 | EU645300 | EU645378 | EU645437 | USNM1027757 | ps |
| *Stylaster horologium* | Southwest Pacific | **707** [228-1163] (6) | | 100 | EU645301 | EU645379 | EU645438 | USNM1071880 | [4] |
| *Stylaster* n. sp. A | Southwest Pacific | **192** (1) | | 100 | EU645302 | EU645380 | EU645439 | USNM1071881 | ps |
| *Stylaster duchassaingi* | West Atlantic | **262** [40-915] (69) | | 97.1 | EU645303 | EU645381 | EU645440 | USNM1078387 | [1] |
| *Stylaster marenzelleri* | Galápagos and Cocos Islands | **342** [88-589] (9) | | 100 | EU645304 | EU645382 | - | USNM84731 | [10,11] |
| *Stylaster galapagensis* | Galápagos and Cocos Islands | **568** [402-806] (13) | | 100 | EU645305 | EU645383 | EU645441 | USNM84716 | [10,11] |
| *Stylaster campylecus* | North Pacific | **194** [23-518] (37) | | 97.3 | EU645306 | EU645384 | EU645442 | USNM1027821 | [7] |
| *Stylaster* cf. *multiplex* | Southwest Pacific | **404** [310-499] (13) | | 100 | EU645307 | EU645385 | EU645443 | USNM1027725 | ps |
| *Stylaster cancellatus* | North Pacific | **346** [152-582] (9) | | 100 | EU645308 | EU645386 | EU645444 | USNM1027822 | [7] |
| *Stylaster polyorchis* | North Pacific | **232** [91-823] (17) | | 100 | EU645309 | EU645387 | EU645445 | USNM1076928 | [7] |
| *Stylaster elassotomus* | North Pacific | **369** [210-881] (7) | | 100 | EU645310 | EU645388 | EU645446 | USNM1027825 | [7] |
| *Stylaster verrillii* | North Pacific | **160** [87-366] (8) | | 100 | EU645311 | EU645389 | EU645447 | USNM1027819 | [7] |
| *Stylaster laevigatus* | West Atlantic | **368** [20-1170] (22) | | 95.5 | EU645312 | EU645390 | - | USNM73189 | [1] |
| *Stylaster imbricatus* | Southwest Pacific | **493** [137-1037] (17)[e] | | 100 | EU645313 | EU645391 | EU645448 | USNM1027748 | [4] |
| *Stylantheca petrograpta* | Northeast Pacific | **3.7** [0-15] (4) | 100 | | EU645327 | EU645398 | EU645455 | USNM1073478 | [7,12,13] |
| *Stylaster californicus* | Northeast Pacific | **39** [12-91] (8)[f] | 75.0 | | EU645314 | EU645392 | EU645449 | USNM1073262 | [7] |
| *Stylaster roseus* | West Atlantic | **84** [2-494] (74)[f] | 67.6 | | EU645315 | EU645393 | EU645450 | USNM1073477 | [1] |
| *Stylaster papuensis* | West Pacific | **13** [6-20] (2) | 100 | | EU645316 | - | EU645451 | USNM1071946 | [5] |
| *Stylaster* sp. D | West Pacific | **9** (1) | 100 | | EU645317 | - | - | USNM1071945 | ps |
| *Stylaster* sp. B | West Pacific | **12** (1) | 100 | | EU645318 | - | EU645452 | USNM1015006 | ps |
| *Stylaster tenisonwoodsi* | West Pacific | **22** [1-90] (5) | 80.0 | | EU645319 | - | - | USNM98511 | [14] |
| *Stylaster* sp. C | West Pacific | **10** (1) | 100 | | EU645320 | - | - | USNM1077817 | ps |
| *Stylaster sanguineus* | Indo-West Pacific | **8** [3-14] (5)[f] | 100 | | EU645321 | - | EU645453 | USNM94601 | [15] |
| *Stylaster erubescens* | West Atlantic | **544** [83-986] (92) | | 100 | EU645322 | EU645394 | - | USNM76610 | [1,3] |
| *Stylaster* cf. *eguchii* | Southwest Pacific | **498** [401-648] (7) | | 100 | EU645323 | EU645395 | EU645454 | USNM1027749 | ps |
| *Stenohelia concinna* | Galápagos Islands | **516** [169-806] (15) | | 100 | EU645324 | EU645396 | - | USNM84747 | [10,11] |
| *Stenohelia pauciseptata* | West Atlantic | **432** [282-514] (3) | | 100 | EU645325 | EU645397 | - | USNM94741 | [1] |
| *Stenohelia profunda* | West Atlantic | **506** [159-2021] (28) | | 100 | EU645326 | - | - | USNM72310 | [1] |
| *Lepidopora glabra* | West Atlantic | **606** [267-1170] (11) | | 100 | EU645328 | - | - | USNM94727 | [1] |
| *Lepidopora microstylus* | Southwest Pacific | **851** [430-1252] (9) | | 100 | EU645329 | EU645399 | EU272644 | USNM1027724 | [4] |
| *Lepidopora* cf. *sarmentosa* | Southwest Pacific | **838** [824-852] (2) | | 100 | EU645330 | EU645400 | EU645456 | USNM1027761 | ps |
| *Lepidopora* n. sp. | Southwest Pacific | **435** (1) | | 100 | EU645331 | EU645401 | EU645457 | USNM1027763 | ps |
| *Lepidopora polystichopora* | Southwest Pacific | **405** [197-710] (9) | | 100 | EU645332 | EU645402 | EU645458 | USNM1027764 | [4] |
| *Lepidopora* n. sp. cf. *polystichopora* | Southwest Pacific | **554** [192-748] (6) | | 100 | EU645333 | EU645403 | EU645459 | USNM1027756 | ps |
| *Lepidotheca* n. sp. cf. *fascicularis* A | Southwest Pacific | **474** (1) | | 100 | EU645334 | EU645404 | EU645460 | USNM1027754 | ps |
| *Lepidotheca* n. sp. cf. *fascicularis* B | Southwest Pacific | **708** (1) | | 100 | EU645335 | EU645405 | EU645461 | USNM1027753 | ps |
| *Lepidotheca macropora* | Galápagos Islands | **464** [169-806] (9) | | 100 | EU645336 | - | - | USNM84699 | [10,11] |
| *Lepidotheca* n. sp. | Southwest Pacific | **562** [427-787] (6) | | 100 | EU645337 | EU645406 | EU645462 | USNM1027727 | ps |

**Table S1.** (continued)

| Species[a] | Geographic Distribution | Bathymetric Distribution (in meters)[b] **Mean** [range] (n) | % Records Shallow (<50m) | Deep (>50m) | GenBank Accession Number 16S | CaM | 18S | Catalog number of specimen used in phylogenetic analyses | Source of distributional records and taxonomy[c] |
|---|---|---|---|---|---|---|---|---|---|
| *Distichopora* n. sp. A | West Pacific | **213** (1) | | 100 | EU645338 | EU645407 | EU645463 | USNM1006516 | ps |
| *Distichopora* cf. *cervina* | West Atlantic | **73** (1) | | 100 | EU645340 | - | - | USNM76603 | ps |
| *Distichopora anceps* | Central Pacific (Hawaii) | **424** [371-539] (8) | | 100 | EU645341 | EU645409 | EU645465 | USNM1021949 | [16,17] |
| *Cyclohelia lamellata* | North Pacific | **190** [83-550] (13) | | 100 | EU645353 | EU645419 | EU645473 | USNM1076490 | [18] |
| *Distichopora borealis* | North Pacific | **261** [79-881] (25) | | 100 | EU645342 | EU645410 | EU645466 | USNM1076507 | [7] |
| *Distichopora asulcata* | Central Pacific (Hawaii) | **326** [293-360] (2) | | 100 | EU645343 | EU645411 | EU645467 | USNM1021956 | [17] |
| *Distichopora irregularis* | West Pacific | **21** [3-46] (11) | 100 | | EU645344 | EU645412 | EU645468 | USNM77033 | [15,19] |
| *Distichopora vervoorti* | West Pacific | **18** (1) | 100 | | EU645345 | EU645413 | - | USNM100001 | [20] |
| *Distichopora* cf. *violacea* | West Pacific | **12** (1) | 100 | | EU645346 | EU645414 | - | USNM87746 | [19] |
| *Distichopora* sp. D | West Pacific | **6** (1) | 100 | | EU645347 | EU645415 | - | USNM1071829 | ps |
| *Distichopora* sp. C | West Pacific | **12** (1) | 100 | | EU645348 | EU645416 | EU645469 | USNM1015004 | ps |
| *Distichopora violacea* | Indo-West Pacific | **6** [0-31] (23) [f] | 100 | | EU645349 | EU645417 | EU645470 | USNM1015005 | ps |
| *Distichopora* sp. B | West Pacific | **7** (1) | 100 | | EU645350 | - | EU645471 | USNM1015007 | ps |
| *Distichopora foliacea* | West Atlantic | **270** [174-507] (23) | | 100 | EU645351 | - | EU645472 | H22-VIII-01-2-N-d | [1] |
| *Distichopora laevigranulosa* | Galápagos Islands | **538** [169-806] (5) | | 100 | EU645352 | EU645418 | - | USNM84707 | [10,11] |
| *Distichopora robusta* | East Pacific | **14** [12-15] (2) | 100 | | EU645339 | EU645408 | EU645464 | USNM1020571 | [21] |
| *Adelopora* n. sp. cf. *fragilis* | Southwest Pacific | **666** [503-1037] (7) | | 100 | EU645354 | EU645420 | EU645474 | USNM1027762 | ps |
| *Adelopora fragilis* | Southwest Pacific | **660** [400-1565] (7) | | 100 | EU645355 | - | - | USNM1071882 | [4] |
| *Adelopora crassilabrum* | Southwest Pacific | **616** [282-1163] (7) | | 100 | EU645356 | EU645421 | EU272642 | USNM1027760 | [4] |
| *Errinopsis fenestrata* | Subantarctic off South America | **377** [165-567] (4) | | 100 | EU645357 | EU645422 | - | USNM83591 | [6] |
| *Errinopora nanneca* | North Pacific | **174** [83-375] (37) | | 100 | EU645358 | EU645423 | EU645475 | USNM1027820 | [7] |
| *Errinopora zarhyncha* | North Pacific | **461** [207-658] (3) | | 100 | EU645359 | - | - | USNM1071915 | [7] |
| *Errina macrogastra* | Galápagos and Cocos Islands | **606** [549-704] (4) | | 100 | EU645360 | - | - | USNM84708 | [10,11] |
| *Inferiolabiata lowei* | Southwest Atlantic and Southwest Pacific | **510** [164-976] (22) | | 100 | EU645361 | EU645424 | EU645476 | USNM1027726 | [4,6] |
| *Lepidotheca chauliostylus* | Southwest Pacific | **782** [130-1265] (10) | | 100 | EU645362 | EU645425 | EU645477 | USNM1027746 | [4] |
| *Stellapora echinata* | Southwest Atlantic | **876** [215-1846] (4) | | 100 | EU645363 | - | - | USNM59945 | [6] |
| *Stephanohelia* n. sp. | Southwest Pacific | **400** [292-528] (8) | | 100 | EU645364 | EU645426 | EU645478 | USNM1073479 | ps |
| *Systemapora ornata* | Southwest Pacific | **428** [285-721] (19) | | 100 | EU645365 | EU645427 | EU645479 | USNM1027729 | [4] |

Abbreviations used are ‘m’ (meters) ‘CAS’ (California Academy of Sciences), ‘H’ (Harbor Branch Oceanographic Institution), ‘USNM’ (United States National Museum of Natural History collection), ‘ps’ (data obtained in the present study);

[a] The list includes 25 undescribed stylasterid species from the Norfolk Ridge in the Southwest Pacific, off New Caledonia (all species referred to by ‘n. sp.’ or ‘n. sp. cf.’ above except *Distichopora* n. sp. A, from off Palau). We use ‘n. sp.’ (*new species*) to refer to a new species that is conspicuously distinct morphologically from any described stylasterid, ‘n. sp. cf.’ (*new species, compare*) to refer a new, undescribed species, to its most morphologically similar congener, and ‘cf.’ (*compare*) to refer a possibly new species to its most morphologically similar congener. Seven tropical shallow-water species sampled in this study (*Distichopora* sp. B, C, D, *Distichopora* cf. *violacea*, and *Stylaster* sp. B, C, D) could not be unambiguously referred to described species and may represent new species. For simplicity, species referred to using ‘n. sp. cf.’ and ‘cf.’ above are referred to solely by ‘cf.’ in Figure 2 and in the main text.

[b] For collecting stations at variable depths, the average between the minimum and maximum depths surveyed in these stations was used for analyses, except for a few specimens collected by submersibles and with depths ranging from zero to over 50 meters (for example, specimen USNM94726, *Stylaster duchassaingi*, with a depth range of ‘0-349 meters’). Since these specimens were not likely collected in shallow-waters by a deep-water submersible, instead of the average depth we used the maximum depth surveyed for analyses;

[c] Distributional records and taxonomy are based on taxonomic work published mostly in the past 25 years and with a comprehensive account of species synonyms and distributions (see sources);

[d] The questionable record of *Conopora verrucosa* at a depth of 5845 meters off Argentina [6] was not used to calculate the depth distribution of the species;

[e] A possible record of *Stylaster imbricatus* at a depth of 4666 to 4731 meters (specimen USNM76719) was not used to calculate the depth distribution of the species;

[f] *Stylaster californicus, Stylaster roseus, Stylaster sanguineus, and Distichopora violacea* are among the most widely observed shallow-water corals, but only a few specimens are deposited in museums. The mean depths calculated herein based on taxonomic revisions and museums records may overestimate the mean depth distribution of these species, particularly for *S. roseus*, whose depth distribution has a mean of 84 meters, but a mode of only 10 meters. The large mean depth calculated for *S. roseus* results from deep-water specimens identified as *S. roseus* in the USNM collection. Since these deep-water specimens differ morphologically from their shallow-water counterparts [1] and may represent a different species, we follow all previous studies (e.g., [1]) and consider *S. roseus* as a shallow-water dweller. One colony of *Stylaster californicus* (USNM43275) and one colony of *S. tenisonwoodsi* (USNM77165) were collected at depths of 91 m and 90 m, respectively, resulting in a relatively deep mean depth for these species. The former species is abundant in shallow-water habitats off California, U.S.A. (e.g., [22]) and may have a depth distribution shallower than the 39 m estimated herein. Shallow-water, Indo-West Pacific species of *Stylaster sanguineus* and *Distichopora violacea* are also abundant and commonly observed in shallow-water tropical reefs. The few museum records available for these species should not be regarded as an indication that they are rare. Shallow-water stylasterids have been collected for over 100 years [23] for jewelry or to be sold to shell collectors, with a harvest of ~9 tons per year solely for *S. californicus* a few decades ago [22]. This practice may have greatly reduced the abundance of stylasterids in shallow waters.

## References


1. Cairns SD (1986) A revision of the Northwest Atlantic Stylasteridae (Coelenterata: Hydrozoa). Smithson Contrib Zool 418: 1-131.
2. Cairns SD (1983) A generic revision of the Stylasterina (Coelenterata: Hydrozoa). Bull Mar Sci 33: 427-508.
3. Zibrowius H, Cairns SD (1992) Revision of the northeast Atlantic and Mediterranean Stylasteridae (Cnidaria: Hydrozoa). Mém Mus Natl Hist Nat 153: 1-136.
4. Cairns SD (1991) The marine fauna of New Zealand: Stylasteridae (Cnidaria: Hydroida). New Zeal Oceanogr Inst Mem 98: 1-180.
5. Zibrowius H (1981) Associations of Hydrocorallia Stylasterina with gall-inhabiting Copepoda Siphonostomatoidea from South-West Pacific, part 1. On the Stylasterine hosts. Bijdr Dierkd 51: 268-286.
6. Cairns SD (1983) Antarctic and Subantarctic Stylasterina (Coelenterata: Hydrozoa). Antarct Res Ser 38: 61-164.
7. Fisher WK (1938) Hydrocorals of the North Pacific Ocean. Proc U S Nat Mus 84: 493-554.
8. Hickson SJ, England HM (1905) The Stylasterina of the Siboga expedition. Siboga-Expeditie 8: 1-26.
9. Boschma H (1968) Notes on the stylasterine coral *Calyptopora pachypoma* (Hickson & England). P K Ned Akad C Biol 71: 315-320.
10. Cairns SD (1986) Stylasteridae (Hydrozoa: Hydroida) of the Galápagos Islands. Smithson Contrib Zool 426: 1-42.
11. Cairns SD (1991) New records of Stylasteridae (Hydrozoa: Hydroida) from the Galápagos and Cocos Islands. P Biol Soc Wash 104: 209-228.
12. Fritchman HK (1974) The planula of the stylasterine hydrocoral *Allopora petrograpta* Fisher: its structure, metamorphosis and development of the primary cyclosystem. Proc 2nd Inter Coral Reef Symp 2: 245-258.
13. Brickmann-Voss A (1996) Seasonality of hydroids (Hydrozoa, Cnidaria) from an intertidal pool and adjacent subtidal habitats at Race Rocks, off Vancouver Island, Canada. Sci Mar 60: 89-97.

14. Cairns SD (1988) New records of Stylasteridae (Cnidaria: Hydrozoa) from Western Australia, including the description of two new species. Rec West Austr Mus 14: 105-119.
15. Boschma H (1957) List of the described species of the order Stylasterina. Zool Verh 33: 1-72.
16. Cairns SD (1978) *Distichopora (Haplomerismos) anceps*, a new stylasterine coral (Coelenterata: Stylasterina) from deep water off the Hawaiian Islands. Micronesica 14: 83-87.
17. Cairns SD (2005) Revision of the Hawaiian Stylasteridae (Cnidaria: Hydrozoa: Athecata). Pac Sci 59: 439-451.
18. Cairns SD (1991) *Cyclohelia lamellata*, new genus and species of Stylasteridae (Cnidaria: Hydrozoa) from the Bering Sea. Pac Sci 45: 383-388.
19. Boschma H (1959) Revision of the Indo-Pacific species of the genus *Distichopora*. Bijdr Dierkd 29: 121-171.
20. Cairns SD, Hoeksema BW (1998) *Distichopora vervoorti*, a new shallow-water stylasterid coral (Cnidaria: Hydrozoa: Stylasteridae) from Bali, Indonesia. Zool Verh 323: 311-318.
21. Lindner A, Cairns SD, Guzman HM (2004) *Distichopora robusta* sp. nov., the first shallow-water stylasterid (Cnidaria: Hydrozoa: Stylasteridae) from the tropical eastern Pacific. J Mar Biol Ass UK 84: 943-947.
22. Gibson ME (1981) The plight of Allopora. Sea Frontiers 27: 211-218.
23. Dall WH (1884) On some Hydrocorallinae from Alaska and California. P Biol Soc Wash 2: 111-115.
