# Supplementary material for: From Offshore to Onshore: Multiple Origins of Shallow-Water Corals from Deep-Sea Ancestors
Source: PLoS One. 2008 Jun 18;3(6):e2429. doi: 10.1371/journal.pone.0002429 (PMC2424010; doi:10.1371/journal.pone.0002429)
Supplement: Table S2 — Fossil stylasterid species and paleontological information. (0.10 MB DOC) [file pone.0002429.s002.pdf]

Table S2. Fossil stylasterid species and paleontological information.

Stage/epoch	Age (mya)	Locality	Paleodepth	Species	Source for taxonomy and paleo-environment
**Deep-water records (offshore)**					
Paleocene (early)	~65	Fakse, Denmark	100-300m	*Astya crassa*[a]<br>*Conopora arborescens*[a]<br>*Errina irregularis*<br>*Errina lobata*<br>*Sporadopora faxensis*[b]<br>*Pliobothrus dispergens*[a]<br>*Pliobothrus laevis*[a]<br>*Congregopora nasiformis*[a]	[1-5]
Eocene (late early)	~50	Seymour Island, Antarctica	100m[c]	*Conopora mariae*	[6]
Eocene (late)	38-42	Eua, Tonga	>200m	*Astya nielseni*<br>*Stylaster sp.* [d]<br>*Crypthelia boschmai*<br>*Crypthelia vetusta*	[1,7]
Miocene (early)	~24.5	Pakaurangi Point, New Zealand	up to 100m at Waiteroa<br>up to 250m at Pakaurangi	*Calyptopora sp. cf. reticulata*	[8,9]
		Pakaurangi Point, New Zealand	up to 300m[e]	*Conopora cf. laevis*	
		Pakaurangi Point, New Zealand	up to 300m[e]	*Inferiolabiata cf. labiata*	
Miocene (lower Messinian)	~6.5	Carboneras, Spain	Upper bathyal	*Calyptopora sp.*<br>*Conopora sp. 1*<br>*Conopora sp. 2*<br>*Conopora sp. 3*<br>*Crypthelia sp. 1*<br>*Crypthelia sp. 2*<br>*Distichopora sp.*<br>*Lepidopora sp.*<br>*Pliobothrus sp. 1*<br>*Pliobothrus sp. 2*<br>*Stenohelia sp.*<br>*Stylaster sp. 1*<br>*Stylaster sp. 2*<br>*Stylaster sp. 3*	[10]
**Shallow-water records (onshore)**					
Oligocene (lower)	32.8-38	Washington state, U.S.A.	shallow-water	*Stylaster milleri*	[11,12]
Pliocene (early)	5.1	Rauhine Range, New Zealand	10-30m[f]	*Sporadopora ?mortenseni*	[8]
Pliocene (early)	3.3-5.1	Kaawa Beach, New Zealand	~5m[f]	*Stylaster gigas*	[8]
Pliocene (early)	5.1	Grange Burn, Australia	shallow-water	*Sporadopora dichotoma*	[13]
Pliocene (Late)	2.0-3.3	Pitt Island, New Zealand	inner shelf	*Sporadopora marginata*	[8,14]
Pliocene (Late)	2.0-3.3	Wanganui, New Zealand	~10-40m	*Sporadopora sp.*	[8,15,16]
Pliocene	3.4	Costa Rica and Panama	37-73m[g]	*Stylaster roseus*	[17]

Table S2. (continued)

Stage/epoch	Locality	Paleodepth	Species	Source for taxonomy and paleo-environment
**Paleodepth undetermined**				
Eocene (Lutetien)	Paris Basin, France	shallow-water?[h]	*Distichopora antiqua*	[3]
Eocene (early-mid)	Kakanui, New Zealand	shallow or deep[i]	*Errina sp.*	[8]
Eocene	Madagascar		*Stylaster sp.*	[12]
Oligocene	Sasselo, North Italy		*Stylaster antiquus*	[3,12]
Oligocene	Lattorf, Central Germany		*Stylaster compressus*	[3,18]
Miocene (early)	Kakanui, New Zealand	Near wavebase?	*Lepidopora sp.*	[7,16]
Miocene	South of Iwaihara, Japan		*Stylater chibaensis*	[19]
Miocene	Moravia, Czech Republic		*Stylaster priscus*	[3,12]
Miocene (middle)	Muddy Creek, Australia		*Distichopora sp.*	[13]
Miocene (middle)	Victoria, Australia		*Stylaster moorabolensis*	[13,18,20]
Pleistocene-Recent	Sicily, Italy		*Stenohelia ?maderensis*	[3]
Pleistocene-Recent	Calabria and Sicily, Italy		*Errina aspera*	[3]
Plio-Pleistocene	Southern Italy		*?Distichopora* sp. [j]	[3]

Abbreviations used are 'mya' (millions of years before present) and 'm' (meters);

[a] Also known from lower Paleocene of Northern Germany [21];

[b] Also known from lower Paleocene (middle and upper Danian, 60.2-63 mya, in paleodepths of 60-80 m) from Paris Basin, France [22];

[c] According to Stolarski [6], some of the formation may have been deposited in shallower waters due to the phenomenon of emergence of deep-sea taxa in high latitudes;

[d] Gastrostyles not observed [7], thus it is possible that *Stylaster* sp. is in fact a *Conopora*;

[e] Paleodepth for most of formation, some thinner units of nearshore and estuarine formation (J. A. Grant-Mackie, personal communication);

[f] Paleodepth estimated by J. A. Grant-Mackie (personal communication);

[g] 'Restricted' depth range, sensu Cairns [17];

[h] 'probably infralittoral' [10]; precise paleodepth not determined. The specimen deposited at the Muséum national d'Histoire naturelle, in Paris, France, was observed by Zibrowius and Cairns [3], who confirm the identification as *Distichopora*;

[i] The presence of several invertebrates indicates deposition in shallow-water, whereas the presence of beds without the foraminifer *Asterocyclina speighti* also suggest deposition in deep waters [14]. The absence of zooxanthellate corals further indicates deep-water deposition (J. A. Grant-Mackie, personal communication);

[j] Specimens not located and possibly based on bryozoans [3].

References

1. Bernecker M, Weidlich O (1990) The Danian (Paleocene) coral limestone of Fakse, Denmark: a model for ancient aphotic, azooxanthellate coral mounds. Facies 22: 103-138.
2. Cairns SD (1983) A generic revision of the Stylasterina (Coelenterata: Hydrozoa). Bull Mar Sci 33: 427-508.
3. Zibrowius H, Cairns SD (1992) Revision of the northeast Atlantic and Mediterranean Stylasteridae (Cnidaria: Hydrozoa). Mém Mus Natl Hist Nat 153: 1-136.
4. Bernecker M, Weidlich O (2005) Azooxanthellate corals in the late Maastrichtian-Early Paleocene of the Danian basin: bryozoan and coral mounds in a boreal shelf setting. In: Freiwald A, Robert JM, editors. Cold-Water Corals and Ecosystems. Berlin: Springer-Verlag, pp. 3-25.
5. Nielsen KB (1919) En Hydrocoralfauna fra Faxe. Medd Dansk Geol Foren 5: 1-65.
6. Stolarski J (1998) *Conopora* (Stylasteridae, Hydrozoa) from the Eocene of Seymour Island. Antarct Sci 10: 487-492.

7. Wells JW (1977) Late Eocene Corals from Eua, Tonga. Geological Survey Professional Paper 640-G.
8. Cairns SD, Grant-Mackie JA (1993) Review of the fossil Stylasteridae (Cnidaria: Hydrozoa) from the New Zealand region. New Zeal J Geol Geophys 36: 1-8.
9. Jones BG (1970) Paleontology and paleoecology of Pakaurangi Point, Kaipara, New Zealand. Trans Roy Soc New Zeal Earth Sci 7: 137-176.
10. Barrier P, Zibrowius H, Lozouet P, Montenat C, Ott d'Estevou P, et al (1991) Une faune de fond dur du bathyal supérieur dans le miocène terminal des Cordillères Bétiques (Carboneras, SE Espagne). Mésogée 51: 3-13.
11. Durham JW (1942) Eocene and Oligocene coral faunas of Washington. J Paleontol 16: 84-104.
12. Boschma H (1951) Notes on Hydrocorallia. Zool Verh 13: 1-49.
13. Hall TS (1898) Stylasteridae from the Victorian Tertiaries. Proc Roy Soc Vict (ns) 10: 175-179.
14. Campbell HJ, Andrews PB, Beu AG, Maxwell PA, Edwards AR, et al (1993) Cretaceous-Cenozoic Geology and Biostratigraphy of the Chatham Islands, New Zealand. Institute of Geological & Nuclear Sciences Monograph 2, Lower Hunt.
15. Fleming CA (1958) The Geology of Wanganui Subdivision. New Zealand Geological Survey, Wellington.
16. Carter RM (1972) Wanganui strata of Komako district, Pohang district, Pohangina valley, Ruahine Range, Manawatu. J Roy Soc New Zeal 2: 293-324.
17. Cairns SD (1999) Stratigraphic distribution of neogene Caribbean azooxanthellate corals (Scleractinia and Stylasteridae). Bull Am Paleontol 357: 109-118.
18. Boschma H (1951) Notes on Stylasterina (Hydrocorallia). P K Ned Akad C Biol 54: 451-458.
19. Eguchi M (1964) A study of Stylasterina from the Antarctic Seas. JARE Scient Rep Ser E 20: 1-100.
20. Hall TS (1893) On two new tertiaty stylasterids. Proc Roy Soc Vict 5: 117-122.
21. Zibrowius H, Voigt E (1993) Ein Fakselkalk-Geschiebe (Danium, Unterpaläozän) aus der Umgebung von Hamburg mit Stylasteriden (Cnidaria: Hydrozoa). Arch Geschiebekde 1: 359-368.
22. Montenat C, Barrier P, Ott d'Estevou P (2002) The Vigny limestones: a record of Paleocene (Danian) tectonic-sedimentary events in the Paris Basin. Sedimentology 49: 421-440.
